# Expedient protocol for myocardial viability assessment with 18 F-FDG positron emission tomography

**DOI:** 10.21203/rs.3.rs-11245740/v1

**Published:** 2026-10-22

**Authors:** Jonathan Ma, Elgin Ozkan, Mohammad Hesam Alavi, Sudhir Jain, Ashwin S Parihar, Thomas Hellmut Schindler

## Abstract

Background We assessed image quality by applying an expedient metabolic preparation period (eMPP) combining simultaneous intravenous insulin and ^18^ F-fluorodeoxyglucose ( ^18^ F-FDG) administration without requiring a specific plasma glucose target for PET myocardial viability imaging in patients with ischemic cardiomyopathy. Methods Expedient MPP for cardiac ^18^ F-FDG uptake was assessed from the time of intravenous infusion of 12.5 or 25 gram of 50% dextrose to the time of ^18^ F-FDG injection. Cardiac ^18^ F-FDG image quality was analyzed according to a 5-point scoring system (5 = excellent to 1 = non-diagnostic) by two independent observers where consensus was achieved in joint reading for cases of disagreement. Further, 58 patients, who underwent the prior standard and MPP (sMPP) for i.v. glucose loading and insulin administration for ^18^ F -FDG PET, served for comparison. Results Eighty-nine consecutive patients (age: 62.5 ± 12.1 yrs, men n = 74 and women n = 15) underwent rest ^99m^ Tc-tetrofosmin SPECT/CT and ^18^ F-FDG PET/CT for the evaluation of myocardial viability. Comparing eMPP and sMPP, the quality scores were excellent in 48% vs. 48%, very good in 26% vs. 24%, good in 16% vs. 10%, fair in 6% vs. 5%, and non-diagnostic in 3% vs. 5%. The mean quality score was not significantly different between eMPP and sMPP (4.10 ± 1.10 vs 4.13 ± 1.17; p = 0.810). As regards the eMPP in diabetic and non-diabetic patients, the quality scores were excellent in 50% vs. 46%, very good in 25% vs. 27%, good in 10% vs. 27%, fair in 10%% vs. 0%, and non-diagnostic in 5% vs. 0%, while the quality score was widely comparable among groups (4.05 ± 1.23 vs 4.18 ± 0.87; p = 0.731). The average MPP was significantly less in the eMPP than in the sMPP group (35 ± 12 vs. 68 ± 19 min; p < 0.0001). Conclusions Using an eMPP after intravenous infusion of dextrose by combining “simultaneous” intravenous insulin and ^18^ F-FDG administration without requiring a specific plasma glucose target, reduces the MPP by 52% compared to prior standard protocol, while maintaining image quality.

